# Improving Classification of Protein Interaction Articles Using Context Similarity-Based Feature Selection

**DOI:** 10.1155/2015/751646

**Published:** 2015-08-03

**Authors:** Yifei Chen, Yuxing Sun, Bing-Qing Han

**Affiliations:** School of Technology, Nanjing Audit University, 86 W. Yushan Road, Nanjing 211815, China

## Abstract

Protein interaction article classification is a text classification task in the biological domain to determine which articles describe protein-protein interactions. Since the feature space in text classification is high-dimensional, feature selection is widely used for reducing the dimensionality of features to speed up computation without sacrificing classification performance. Many existing feature selection methods are based on the statistical measure of document frequency and term frequency. One potential drawback of these methods is that they treat features separately. Hence, first we design a similarity measure between the context information to take word cooccurrences and phrase chunks around the features into account. Then we introduce the similarity of context information to the importance measure of the features to substitute the document and term frequency. Hence we propose new context similarity-based feature selection methods. Their performance is evaluated on two protein interaction article collections and compared against the frequency-based methods. The experimental results reveal that the context similarity-based methods perform better in terms of the *F1* measure and the dimension reduction rate. Benefiting from the context information surrounding the features, the proposed methods can select distinctive features effectively for protein interaction article classification.

## 1. Introduction

An overwhelming number of biological articles are published daily online as a result of growing interest in biological research, especially relating to the study of protein-protein interactions (PPIs). It is essential to classify which articles describe PPIs, that is, to filter out those irrelevant articles from the whole collection of the biological literature. This allows a more efficient extraction of PPIs from the large amount of biological literature. Automated text classification is a key technology to rapidly find relevant articles. Text classification has been successfully applied to various domains such as text sentinel classification [1], spam e-mail filtering [2, 3], author identification [4], and web page classification [5]. Research on protein interaction article classification (IAC) is a text classification task with practical significance in the biological domain.

In the classic text classification framework, a feature extraction mechanism extracts features from raw articles, including all distinct terms (words). This is also known as bag-of-words (BOW) representation for text documents. Hence each article is represented by a multidimensional feature vector where each dimension corresponds to a term (feature) within the literature collection. Even a small literature collection would contain tens of thousands of features [6, 7]. The high dimensionality of the feature space not only increases computational time but also degrades classification performance. Hence, automated feature selection plays an essential role in making the text classification more efficient and accurate by selecting a subset of the most important features [8, 9]. Feature selection is an active research area in many fields such as data mining, machine learning, and rough sets [10–13].

The process of feature selection typically involves certain metrics that are designed for measuring the importance level of features, and the most important features are selected to help in efficient utilization of resources for large scale problems [14]. The existing feature selection methods are mostly based on the statistical information in documents, including term frequency and document frequency [7, 14–18]. Term frequency is the number of times a particular term appears in a document while document frequency is the number of documents containing that term within the literature collection. One potential drawback of most of these frequency-based feature selection methods is that they treat each feature separately [19]. In other words, these approaches are context independent: they do not utilize the context information around the terms when judging their importance, such as word order, word cooccurrence, multiword chunks, and semantic relationships. However, this information is important for classifying which articles are PPI relevant or nonrelevant. For example, protein names exist in both PPI relevant and nonrelevant documents. So they could have great document frequency or term frequency. However, obviously they are not distinctive terms for the purpose of classification. Hence, it is difficult to measure the importance of all the terms just according to the document frequency or term frequency. After in-depth research we have noticed that, in the PPI relevant documents, the fact that proteins interact with each other is described through the context of those proteins. Meanwhile in nonrelevant documents, the fact that there are no interactions between the particular proteins is also depicted within the context of the documents. The above observation leads us to an interesting issue which is that the context of features in biological articles can be utilized to measure feature importance and to improve the feature selection process. Hence we propose context similarity-based feature selection methods.

This paper is organized as follows: we provide an overview of the existing frequency-based feature selection methods for text classification in Section 2, and this is followed by a definition of the proposed context similarity-based feature selection methods. Then in order to examine the two kinds of methods carefully, the experimental results and discussion are presented in Section 3 to find which one is more useful in the IAC task. This is followed by a conclusion in Section 4.

## 2. Materials and Methods

### 2.1. Existing Feature Selection Methods for Text Classification

Feature selection is a process which selects a subset of the most important features. Such selection can help in building effective and efficient models for text classification. Normally, feature selection techniques can be divided into three categories: filters, wrappers, and embedded methods [19]. Filters measure feature importance using various scoring metrics that are independent of a learning model or classifier and select top-*N* features attaining the highest scores. Univariate filter techniques are computationally fast. However, they do not take feature dependencies into consideration, which was discussed as the motivation of this paper in Section 1. In addition, multivariate filter techniques incorporate feature dependencies to some degree, while they are slower and less scalable than univariate techniques. Wrappers evaluate features using a certain search algorithm together with a specific learning model or classifier. Wrapper techniques consider feature dependencies and provide interaction between features during the subset search processing but are computationally expensive compared with filters. Embedded methods integrate feature selection into the model learning phase. Therefore, they merge with the model or classifier much further than the wrappers. Nevertheless, they are also computationally more intensive than filters.

Considering the high dimensionality of the feature space for text classification tasks, the most frequently used approach for feature selection is the univariate filter method [7]. And among them four document frequency-based methods and two term frequency-based methods that will be discussed in the paper are illustrated as follows, where *P*(*t*_*k*_∣*c*_*i*_) is the percentage of documents belonging to a category *c*_*i*_ in which the term *t*_*k*_ occurs and $P\left(t_{k}|{\bar{c}}_{i}\right)$ is the percentage of documents not belonging to a category *c*_*i*_ in which the term *t*_*k*_ occurs. |*c*| is the number of categories, which is 2 for the IAC task.

(*1) Document Frequency (DF)*. Document frequency (DF) is a simple and effective feature selection method which is based on the assumption that infrequent terms are not reliable in text classification and may degrade the performance [7]. Hence, if the document frequency in which a term occurs is the largest, the term is retained [20]. The DF metrics of the term *t*_*k*_ can be computed as follows:

(1) $$\begin{array}{c} DF\left(t_{k}\right)=\overset{\left|c\right|}{\underset{i=1}{\sum }}DF\left(t_{k},c_{i}\right)=\overset{\left|c\right|}{\underset{i=1}{\sum }}P\left(t_{k}\;|\;c_{i}\right), \end{array}$$

where DF(*t*_*k*_, *c*_*i*_) is the DF measure of the term *t*_*k*_ in a category *c*_*i*_ and DF(*t*_*k*_) is the sum of DF(*t*_*k*_, *c*_*i*_) across all the categories. 

(*2) Gini Index (GI)*. Gini Index (GI) was originally used to find the best attributes in decision trees. Shang et al. [15] proposed an improved version of the GI method to apply it directly to text feature selection. The GI(*t*_*k*_, *c*_*i*_) measures the purity of the feature *t*_*k*_ towards a category *c*_*i*_. Its sum across categories, GI(*t*_*k*_), is given as

(2) $$\begin{array}{c} GI\left(t_{k}\right)=\overset{\left|c\right|}{\underset{i=1}{\sum }}GI\left(t_{k},c_{i}\right)=\overset{\left|c\right|}{\underset{i=1}{\sum }}P{\left(t_{k}\;|\;c_{i}\right)}^{2}P{\left(c_{i}\;|\;t_{k}\right)}^{2}, \end{array}$$

where *P*(*c*_*i*_∣*t*_*k*_) is the conditional probability of the feature *t*_*k*_ belonging to a category *c*_*i*_ given presence of the feature *t*_*k*_. 

(*3) Class Discriminating Measure (CDM)*. Class discriminating measure (CDM) is a derivation of the odds ration introduced by Chen et al. [16]. The results in their paper indicate that CDM is a better feature selection approach than information gain (IG). The CDM calculates the effectiveness of the term *t*_*k*_ as follows:

(3) $$\begin{array}{c} CDM\left(t_{k}\right)=\overset{\left|c\right|}{\underset{i=1}{\sum }}CDM\left(t_{k},c_{i}\right)=\overset{\left|c\right|}{\underset{i=1}{\sum }}\left|log\frac{P\left(t_{k}\;|\;c_{i}\right)}{P\left(t_{k}\;|\;{\bar{c}}_{i}\right)}\right|, \end{array}$$

where CDM(*t*_*k*_, *c*_*i*_) is the CDM measure of the term *t*_*k*_ in a category *c*_*i*_ and CDM(*t*_*k*_) is the sum of CDM(*t*_*k*_, *c*_*i*_) across all the categories. 

(*4) Accuracy Balanced (Acc2)*. Accuracy balanced (Acc2) is a two-side metric (it selects both negative and positive features) that is based on the difference of the distributions of a term belonging to a category and not belonging to that category in the documents. In Forman [14], the Acc2 is studied and claimed to have a performance comparable to the IG and chi-square statistical metrics. The Acc2 of the term *t*_*k*_ can be computed as follows:

(4) $$\begin{array}{c} Acc2\left(t_{k}\right)=\overset{\left|c\right|}{\underset{i=1}{\sum }}Acc2\left(t_{k},c_{i}\right)=\overset{\left|c\right|}{\underset{i=1}{\sum }}\left|P\left(t_{k}\;|\;c_{i}\right)-P\left(t_{k}\;|\;{\bar{c}}_{i}\right)\right|, \end{array}$$

where Acc2(*t*_*k*_, *c*_*i*_) is the Acc2 measure of the term *t*_*k*_ in a category *c*_*i*_ and Acc2(*t*_*k*_) is the sum of Acc2(*t*_*k*_, *c*_*i*_) across all the categories.

(*5) Term Frequency Inverse Document Frequency (TFIDF)*. Term frequency inverse document frequency (TFIDF) is a numerical statistic that is intended to reflect how important a term is to a document in a collection or corpus. One of the simplest filter metrics is computed by summing the TFIDF. Wei et al. [21] introduced category information to TFIDF, which can be reformed using a notation of term frequency tf(*t*_*k*_, *c*_*i*_) that is the number of occurrences of a term *t*_*k*_ in documents from a category *c*_*i*_. Consider

(5) $$\begin{array}{c} TFIDF\left(t_{k}\right)=\overset{\left|c\right|}{\underset{i=1}{\sum }}tf\left(t_{k},c_{i}\right)\times log\left(\frac{1}{P\left(t_{k}\;|\;c_{i}\right)}\right). \end{array}$$

(*6) Normalized Term Frequency-Based Gini Index (GINI*_*NTF*_). Normalized term frequency-based Gini Index (GINI_NTF_) revised the document frequency in the Gini Index metric with the term frequency by Azam and Yao [17]. Experimental results revealed that the term frequency-based metric was useful in feature selection. We reform the formula of GINI_NTF_ as follows:

(6) GININTFtk=∑i=1|c|tfnormtk,cidocci2 ×tfnormtk,citfnormtk,ci+tfnormtk,c¯i2,

where tf_norm_(*t*_*k*_, *c*_*i*_) is the normalized term frequency of *t*_*k*_ in documents from a category *c*_*i*_ and $tf_{norm}(t_{k},{\bar{c}}_{i})$ is the normalized term frequency of *t*_*k*_ in documents not from a category *c*_*i*_. The normalized values of term frequency are used in the metric so that term frequencies are not influenced by varying lengths of documents.

### 2.2. Context Similarity-Based Feature Selection Methods

According to the bag-of-words document representation, each raw document in the article collection is transformed into a high-dimensional vector before the process of text classification. In order to address the issues of high dimensionality, the feature filter methods, such as the DF, GI, CDM, and Acc2, are utilized to select the most important features based on document frequency. One potential problem of these frequency-based methods is that they ignore the context relationships between features. As we have discussed in Section 1, context information is essential for the IAC task. When attempting to judge the importance levels of features, it may be advantageous to explicitly compare the similarity shared among contexts in PPI relevant articles or nonrelevant articles. Hence when building the feature selection metrics, we take the significance of context information of each feature into account through the context similarity.

*Context Similarity Measure*. sim_context_(*t*_*k*_, *c*_*i*_) is designed to explicitly express the similarity shared by contexts of the term *t*_*k*_ in a certain category *c*_*i*_. The measure is based on the word cooccurrences and chunks of a pair of context strings context_*d*_(*t*_*k*_, *w*) and context_*d*′_(*t*_*k*_, *w*) containing the term *t*_*k*_ within a category *c*_*i*_. context_*d*_(*t*_*k*_, *w*) denotes a document *d* containing a term *t*_*k*_ within a context string {*t*_−*wk*_,…, *t*_−1*k*_, *t*_*k*_, *t*_1*k*_,…, *t*_*wk*_}, where *w* is a window size that takes into account *w* terms before and after the term *t*_*k*_. The term *t*_*k*_ is contained in another context string of document *d*′, context_*d*′_(*t*_*k*_, *w*), which is {*t*_−*wk*_′,…, *t*_−1*k*_′, *t*_*k*_, *t*_1*k*_′,…, *t*_*wk*_′} with the window size *w*. Using context_*d*_, a multiword phrase chunk containing *t*_*k*_ and its word cooccurrence can be considered to measure the importance of *t*_*k*_.

First sim(context_*d*_(*t*_*k*_, *w*), context_*d*′_(*t*_*k*_, *w*)) is defined to measure the similarity between the context string pair (context_*d*_, context_*d*′_) as follows:

(7) simcontextdtk,w,contextd′tk,w=∑w=0wdiscontextdtk,w,contextd′tk,ww+1.

The sum of all the context strings from 0 to maximum window size |*w*| is utilized to incorporate word cooccurrence and phrase similarity comprehensively. |*w*| is used to control the scope of the local information of term *t*_*k*_ involved in the measurement, and trials on the training data show that |*w*| = 3 is the optimal value. In this paper, Jaro-Winkler [22] distance is employed as the distance function of two context strings, dis(context_*d*_(*t*_*k*_, *w*), context_*d*′_(*t*_*k*_, *w*)), because it was designed and best suited for short strings. The Jaro-Winkler distance is a measure of similarity between two strings, and it is a variant of the Jaro distance metric [23, 24]. The higher the Jaro-Winkler distance for two strings is, the more similar the strings are. The score is normalized such that 0 equates to no similarity and 1 is an exact match.

Then, sim_context_(*t*_*k*_, *c*_*i*_) is defined to measure the similarity of context in the documents containing the term *t*_*k*_ belonging to a category *c*_*i*_ as follows:

(8) simcontexttk,ci=∑contextd,contextd′∈cksimcontextdtk,w,contextd′tk,w.

*Context Similarity-Based Feature Selection Methods*. In order to elaborate the context similarity-based feature selection metrics, the class discriminating measure (CDM) is considered as an example, which was very useful in reducing the feature set in some application domains. The metric of CDM has been defined in Section 2.1 based on *P*(*t*_*k*_∣*c*_*i*_) and $P\left(t_{k}|{\bar{c}}_{i}\right)$. Here *P*(*t*_*k*_∣*c*_*i*_), the percentage of documents with the term *t*_*k*_ belonging to the category *c*_*i*_, can also be represented as doc(*t*_*k*_, *c*_*i*_)/doc(*c*_*i*_), where doc(*t*_*k*_, *c*_*i*_) is the document frequency containing the term *t*_*k*_ in the category *c*_*i*_ and doc(*c*_*i*_) is the total number of articles in the category *c*_*i*_. $P\left(t_{k}|{\bar{c}}_{i}\right)$, the percentage of documents with the term *t*_*k*_ not belonging to the category *c*_*i*_, can be represented as $doc(t_{k},{\bar{c}}_{i})/doc({\bar{c}}_{i})$, where $doc(t_{k},{\bar{c}}_{i})$ is the document frequency containing the term *t*_*k*_ not in the category *c*_*i*_ and $doc({\bar{c}}_{i})$ is the total number of articles not in the category *c*_*i*_. Hence, we can have the following CDM metric:

(9) CDMtk=∑i=1clogPtk ∣ ciPtk ∣ c¯i=∑i=1clogdoctk,cidocci·docc¯idoctk,c¯i.

In order to make use of the context information of terms and not just the document frequency, we substitute the context similarity measure sim_context_(*t*_*k*_, *c*_*i*_) for the document frequency doc(*t*_*k*_, *c*_*i*_). Then the obtained metric with reformed definition is referred to as CDM_cs_, class discriminating measure based on context similarity. If the context similarity of a term within a certain text category is greater, the term is more important for text classification. The definition of CDM_cs_ is as follows:

(10) $$\begin{array}{c} CDM_{cs}\left(t_{k}\right)=\overset{\left|c\right|}{\underset{i=1}{\sum }}\left|log\left(\frac{sim_{context}\left(t_{k},c_{i}\right)}{doc\left(c_{i}\right)}\cdot \frac{doc\left({\bar{c}}_{i}\right)}{sim_{context}\left(t_{k},{\bar{c}}_{i}\right)}\right)\right|. \end{array}$$

The other three document frequency-based metrics defined in Section 2.1 can also be reformed in the same way based on the context similarity to Acc2_cs_, GI_cs_, and DF_cs_:

(11) $$\begin{array}{c} Acc2_{cs}\left(t_{k}\right)=\overset{\left|c\right|}{\underset{i=1}{\sum }}\left|\frac{sim_{context}\left(t_{k},c_{i}\right)}{doc\left(c_{i}\right)}-\frac{sim_{context}\left(t_{k},{\bar{c}}_{i}\right)}{doc\left({\bar{c}}_{i}\right)}\right|,\\ GI_{cs}\left(t_{k}\right)=\overset{\left|c\right|}{\underset{i=1}{\sum }}\left|{\left(\frac{sim_{context}\left(t_{k},c_{i}\right)}{doc\left(c_{i}\right)}\right)}^{2}{\left(\frac{sim_{context}\left(t_{k},c_{i}\right)}{doc\left(t_{k}\right)}\right)}^{2}\right|,\\ DF_{cs}\left(t_{k}\right)=\overset{\left|c\right|}{\underset{i=1}{\sum }}\frac{sim_{context}\left(t_{k},c_{i}\right)}{doc\left(c_{i}\right)}, \end{array}$$

where doc(*t*_*k*_) is the number of documents containing the term *t*_*k*_ in all the text categories.

## 3. Results and Discussion

### 3.1. Experimental Settings

*Classification Model Model*_*SVM*_*poly*_. Support vector machines (SVMs) pioneered by Vapnik [25] are suitable for complex classification problems. Their power comes from the combination of the kernel trick and maximum margin hyperplane separation. SVMs are one of the most successful approaches for classification in text mining [26, 27]. Hence, in this paper, we employ the SVMs with a polynomial kernel as a classification model, Model_SVM_poly_, which is trained and tested using the LIBSVM toolbox [28]. A 10-fold cross-validation is adopted to tune parameters.

*Data Sets*. An in-depth investigation will be carried out to compare the performances of the four proposed context similarity-based methods and the six existing frequency-based feature selection methods. Two data sets (Data_BCII_ and Data_BCIII_) are used in our experiments to evaluate the performance, which are both extracted from the BioCreAtIvE (the Critical Assessment of Information Extraction in Biology) challenges. The challenges were set up to evaluate the state of the art of text mining and information extraction in the biological domain.

In the data preprocessing step, all words are converted to lower case, punctuation marks and stop words are removed, and no stemming is used. Consider the following.

(1) Data_BCII_: we obtain the Data_BCII_ from the Protein Interaction Article Subtask (IAS) of the BioCreAtIvE II challenge [29]. The Data_BCII_ is composed of abstracts of 6,172 articles in total, which are taken from a set of MEDLINE articles that are annotated as interaction articles or not according to the guidelines used by the MINT and IntAct databases. There are 5,495 abstracts used as training data and 677 ones as test data. And there are 3,536 and 338 interaction articles, that is, positive examples, in the training and test set, respectively.
(2) Data_BCIII_: we obtain the Data_BCIII_ from the PPI Article Classification Task (ACT) of the BioCreAtIvE III challenge [30]. The training set (TR) consists of a balanced collection of 2,280 articles classified through manual inspection, divided into PPI relevant and nonrelevant articles. The annotation guidelines for this task were refined iteratively based on the feedback from both annotation databases and specially trained domain experts. The development (DE) and test (TE) set take into account PPI relevant journals based on the current content of collaborating PPI databases. Random samples of abstracts from these journals were taken to generate a development set of 4,000 abstracts (628 PPI relevant and 3,318 nonrelevant abstracts) in total and a test set of 6,000 abstracts (918 PPI relevant and 5,090 nonrelevant abstracts). These two disjointed sets were drawn from the same sample collection.

*Performance Measures*. Since the applications are restricted to IAC, which is a binary classification task, we measure the performance in terms of *F*1 measure [20]. The *F*1 is determined by a combination of precision and recall. Precision is the percentage of documents that are correctly classified as being positive. Recall is the percentage of positive documents that are correctly classified. The precision, recall, and *F*1 are obtained as

(12) $$\begin{array}{c} Precision=\frac{TP}{TP+FP},\\ Recall=\frac{TP}{TP+FN},\\ F1=\frac{2\times \text{Precision}\times Recall}{Precision+Recall}, \end{array}$$

where TP is the number of positive documents that are correctly classified as positive ones, FP is the number of negative documents that are misclassified as positive ones, TN is the number of negative documents that are correctly classified as negative ones, and FN is the number of positive documents that are misclassified as negative ones.

### 3.2. Experimental Results on the Data_BCII_

**Figure 1:**
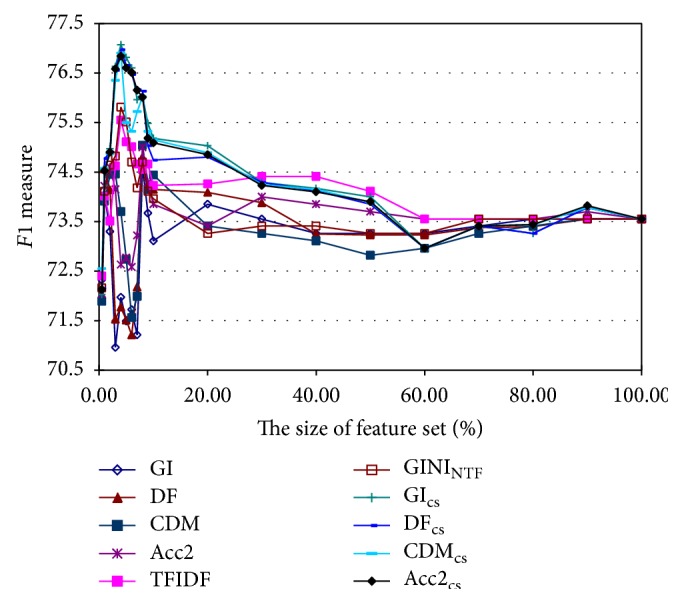
The *F*1 performance curves of all the feature selection methods on the Data_BCII_ when the number of the selected features is the top 0.5%, 1%, 2%, 3%, 4%, 5%, 6%, 7%, 8%, 9%, 10%, 20%, 30%, 40%, 50%, 60%, 70%, 80%, 90%, and 100%.

First, we test all the feature selection methods when Model_SVM_poly_ is applied on the Data_BCII_ data set, where there are 29,979 total features extracted using the bag-of-words document representation. The proposed context similarity-based methods, GI_cs_, DF_cs_, CDM_cs_, and Acc2_cs_, are compared with the frequency-based methods, GI, DF, CDM, Acc2, TFIDF, and GINI_NTF_, when the number of the selected features is the top 0.5%, 1%, 2%, 3%, 4%, 5%, 6%, 7%, 8%, 9%, 10%, 20%, 30%, 40%, 50%, 60%, 70%, 80%, 90%, and 100%.  Figure 1 shows the trend curves of all the feature selection methods, and the optimal parameter value of the window size of context information is 3, which is tuned through 10-fold cross-validation.

Figure 1 indicates that all these feature selection methods have a similar trend on the Data_BCII_, and the proposed methods are more effective. The context similarity-based methods and the term frequency-based methods achieve the best performance when around 4% top important features are selected, while the document frequency-based methods obtain the best performance when around 7-8% features are used. Moreover, the proposed methods outperform the other methods on selecting the top important features to achieve the best *F*1 measure. Among the context similarity-based feature selection methods, when the top 1300 features (4.3% of total number of features) are selected, GI_cs_ acquires the highest *F*1 measure 77.07, which effectively improves the *F*1 measure of the Model_SVM_poly_ when all the features are used (73.55) by 3.52.

Further, in order to study the performance of all these feature selection methods in more detail, a small feature set in the scope of the top 2000 is used. The corresponding *F*1 measure results are shown in Table 1 when the top 100, 300, 500, 700, 900, 1100, 1300, 1500, 1700, and 1900 features are selected. The best result for each feature set is shown in bold. It can be seen from Table 1 that the context similarity-based methods outperform those methods based on the document frequency or term frequency. The last column of Table 1 presents the best performance of the Model_SVM_poly_ that various feature selection methods can achieve, and the size of selected features when the best performance is achieved is illustrated in the parentheses. It can be seen that, compared with the four document frequency-based methods, the TFIDF and the GINI_NTF_ perform better, which shows that term frequency is a relatively more important factor than document frequency. Moreover, all the context similarity-based methods achieve better performance with fewer selected features, and among them the GI_cs_ performs the best on the Data_BCII_. Hence, the proposed method can extract more effective information from context similarity measure of term cooccurrences and chunks than just calculating the document frequency or term frequency. This context information is helpful when measuring the importance of features to boost the performance.

### 3.3. Experimental Results on the Data_BCIII_

**Figure 2:**
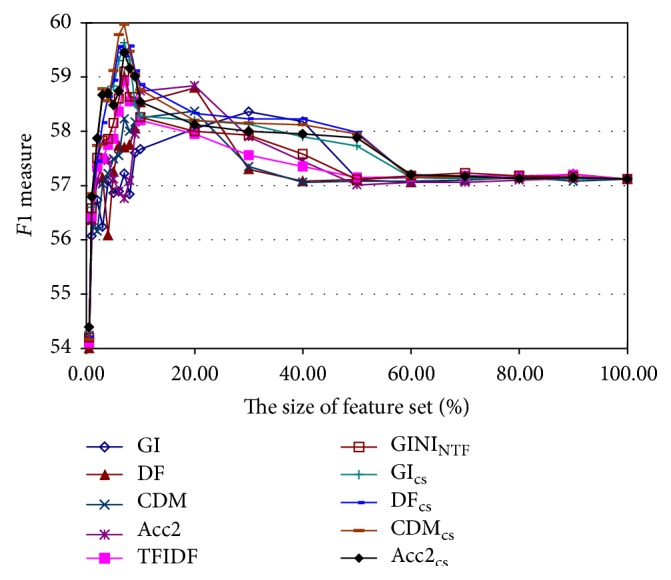
The *F*1 performance curves of all the feature selection methods on the Data_BCIII_ when the number of selected features is the top 0.5%, 1%, 2%, 3%, 4%, 5%, 6%, 7%, 8%, 9%, 10%, 20%, 30%, 40%, 50%, 60%, 70%, 80%, 90%, and 100%.

Then, we test the proposed feature selection methods on the Data_BCIII_ when the number of selected features is the top 0.5%, 1%, 2%, 3%, 4%, 5%, 6%, 7%, 8%, 9%, 10%, 20%, 30%, 40%, 50%, 60%, 70%, 80%, 90%, and 100%, where there are 23,084 features extracted using the bag-of-words representation in total.  Figure 2 shows the trend curves of the *F*1 measure versus different sizes of selected features. From Figure 2 we can see that when around 7% top important features are used, the proposed methods and term frequency-based methods can achieve the best performance, while document frequency-based methods need to utilize more than 15% top features to achieve their best performance, which is less effective.

Then, for the purpose of more detailed study on a small feature set, Table 2 shows the *F*1 measure results when the size of the selected features is 100, 300, 500, 700, 900, 1100, 1300, 1500, 1700, and 1900. The best result for each feature set is shown in bold. It can be seen that on the Data_BCIII_ the performance of the context similarity-based methods is also better than that of their corresponding frequency-based methods. And when the size of the feature set is 1700 (7.4% of the total number of features), CDM_cs_ acquires the highest *F*1 measure value 59.97, which improves the *F*1 measure of the Model_SVM_poly_ when all the features are used (57.12) by 2.85. Hence the context information of terms is helpful for the feature selection in IAC applications.

We notice that there is a significant drop in performance from the Data_BCII_ to Data_BCIII_, which suffered from the fact that the training article collection is extracted from different online article sources compared with the test data sets, and that the test data sets have the high class skew problem [30].

### 3.4. Analysis and Discussion

*Comparison of the Selected Features*. Besides the *F*1 measure results, we also analyze the effectiveness of feature selection methods through studying the profile of the selected features. The sorted lists of the top-10 features picked by each method are given in Tables 3 and 4 on the Data_BCII_ and Data_BCIII_, respectively. The features that are selected commonly by all the methods are indicated in bold. These common features make the same contribution to the classification performance, such as “interact” in Table 3 and “interaction” in Table 4. Hence we compare the special features selected by different methods. We note that there are two categories of special selected features according to two different feature selection principals. The first category features are the ones selected based on the statistical frequency. These features obtain higher scores because more documents contain them or they occur more. However, the term cooccurrences and chunks within the document are ignored. For example, the terms “protein” and “cell” are selected by all the frequency-based methods but the context similarity-based methods on both Data_BCII_ and Data_BCIII_. Considering “protein,” it is just used to describe different protein names, which can appear anywhere in biological articles with the result of high document frequency or term frequency. However, it is not a distinctive feature to classify PPI relevant or nonrelevant articles. If such irrelevant features are assigned higher scores by a feature selection method, the performance obtained by those features would be degraded. On the contrary, these features are assigned lower values by our proposed methods, because their context dissimilarity between the PPI relevant and nonrelevant articles depresses their scores. The second category features are shared by the context similarity-based methods, such as the terms “activate” in Table 3 and “activity” in Table 4. Their evaluation scores are raised by the context similarity within the PPI relevant articles, which is important for the classification purpose.

In order to further study the proposed methods on common and special selected features, the top 1000 features are selected on both data sets, respectively. We perform experiments on the pairs of one context similarity-based method and one frequency-based feature selection method. First, the common features selected for each pair by both feature selection methods are fed into the Model_SVM_poly_. Then the performance of this Model_SVM_poly_ based on the common features is compared with the performance achieved based on all the top 1000 features selected by the context similarity-based method and the frequency-based method, respectively. Our purpose is to reveal which kind of feature selection methods can increase the performance more with their special selected features. The results are listed in Tables 5 and 6 on the Data_BCII_ and Data_BCIII_, respectively. It can be seen that the increments of context similarity-based methods are higher than the frequency-based methods, so the special features selected through context similarity-based methods can bring more distinctive information for the classifier on both data sets.

*Dimension Reduction Rate*. In addition to *F*1 measure, dimension reduction rate is another important aspect of feature selection. Therefore, a dimension reduction is also studied during the experiments. To compute a dimension reduction rate together with the *F*1 measure, a scoring scheme from Gunal and Edizkan [31] is defined as follows:

(13) $$\begin{array}{c} Score=\frac{1}{k}\overset{k}{\underset{i=1}{\sum }}\frac{{dim}_{N}}{{dim}_{i}}F1_{i}, \end{array}$$

where *k* is the number of trails, dim_*N*_ is the maximum feature size, dim_*i*_ is the feature size at the *i*th trail, and *F*1_*i*_ is the *F*1 measure of the *i*th trail. Here, dim_*i*_ is a set of sequences, 100, 300, 500, 700, 900, 1100, 1300, 1500, 1700, and 1900, and so *k* is 10. The results of dimension reduction analysis using the described scoring scheme are presented in Table 7. It is apparent from this table that the context similarity-based feature selection methods provide better performance than the frequency-based methods.

## 4. Conclusions

In this paper, novel context similarity-based feature selection methods were introduced for text classification in the biological domain to classify protein interaction articles. They assign importance scores to features based on their similarity measure of context information within certain text categories. Using two different data sets, the performance of the proposed methods was investigated and compared against four document frequency-based and two term frequency-based methods. The effectiveness of the proposed methods was demonstrated and analyzed on the *F*1 measure, the profile of selected features, and dimension reduction rate for the IAC tasks. Since IAC is a binary text classification task in biological domain, we also want to know the performance of the proposed methods when they are extended to multiclass problems. Hence, an adaptation of the context similarity-based selection method to multiclassification problems remains an interesting future task.

## Figures and Tables

**Table 1:** The *F*1 measure results when the Model_SVM_poly_ is applied to the Data_BCII_ when the top 100, 300, 500, 700, 900, 1100, 1300, 1500, 1700, and 1900 features are selected. In each column, the bold value indicates the best performance for each feature set when various feature selection methods are used, respectively. The “best” column presents the best performance that various feature selection methods can achieve, and the numbers in the parentheses are the corresponding sizes of feature sets.

Number of features	100	300	500	700	900	1100	1300	1500	1700	1900	best
GI	72.32	74.04	73.30	73.34	70.96	71.79	71.97	71.49	71.27	71.21	74.60(2300)
DF	72.02	74.56	74.14	72.50	71.53	72.21	71.77	71.51	71.21	72.18	74.80(2300)
CDM	71.89	73.91	74.45	74.49	74.46	74.25	73.70	72.73	71.56	71.99	75.04(2100)
Acc2	72.40	74.22	74.60	73.49	74.15	74.01	72.63	72.77	72.58	73.22	75.00(2100)
TFIDF	72.40	74.01	73.51	73.61	74.62	75.11	75.55	75.11	75.01	74.66	75.55(1300)
GINI_NTF_	72.16	74.11	74.64	74.70	74.82	75.41	75.81	75.51	74.70	74.18	75.81(1300)
GI_cs_	72.03	74.52	**74.97**	**76.14**	**76.63**	**76.66**	**77.07**	**76.81**	**76.60**	75.96	**77.07**(1300)
DF_cs_	72.15	**74.77**	74.90	76.09	76.55	76.60	76.97	76.65	76.47	**76.16**	76.97(1300)
CDM_cs_	**72.55**	74.57	74.87	75.99	76.35	76.47	76.90	75.50	75.38	75.81	76.90(1300)
Acc2_cs_	72.13	74.53	74.90	76.06	76.58	76.61	76.84	76.60	76.51	76.15	76.84(1300)

**Table 2:** The *F*1 measure results when the Model_SVM_poly_ is used on the Data_BCIII_ when the top 100, 300, 500, 700, 900, 1100, 1300, 1500, 1700, and 1900 features are selected. In each column, the bold value indicates the best performance for each feature set when various feature selection methods are used, respectively. The “best” column presents the best performance that various feature selection methods can achieve, and the numbers in the parentheses are the corresponding sizes of feature sets.

Number of features	100	300	500	700	900	1100	1300	1500	1700	1900	best
GI	52.16	56.07	56.73	56.24	57.08	56.86	56.89	57.34	57.22	56.84	58.36(5900)
DF	50.91	56.37	57.42	57.14	56.98	57.25	57.73	57.09	57.70	57.75	58.80(4300)
CDM	52.13	56.43	56.16	57.03	57.22	57.49	57.55	58.27	58.23	58.00	58.37(4500)
Acc2	52.24	56.35	57.07	57.48	57.46	57.12	56.89	57.14	56.76	57.09	58.84(3900)
TFIDF	52.10	56.43	57.34	57.50	57.75	57.86	58.26	58.51	58.93	58.55	58.93(1700)
GINI_NTF_	52.20	56.58	57.51	57.83	57.86	58.05	58.60	58.83	59.10	58.63	59.10(1700)
GI_cs_	52.30	56.62	57.80	58.66	58.76	58.83	59.30	59.51	59.63	59.31	59.63(1700)
DF_cs_	52.21	56.80	57.45	58.16	58.64	58.94	59.56	59.39	59.39	**59.57**	59.57(1900)
CDM_cs_	52.17	**56.85**	57.74	**58.78**	**59.06**	**59.12**	**59.78**	**59.81**	**59.97**	59.47	**59.97**(1700)
Acc2_cs_	**52.39**	56.79	**57.87**	58.67	58.70	58.48	58.74	59.06	59.45	59.16	59.45(1700)

**Table 3:** The top 10 features on the Data_BCII_ selected by various feature selection methods. The terms that are selected commonly by all the methods are indicated in bold.

Number	GI	DF	CDM	Acc2	TFIDF	GINI_NTF_	GI_cs_	DF_cs_	CDM_cs_	Acc2_cs_
1	protein	protein	hybrid	bind	proteins	protein	**interact**	**interact**	bind	**interact**
2	bind	bind	**interact**	interaction	cell	bind	bind	hybrid	**interact**	hybrid
3	interaction	proteins	protein	domain	receptor	domain	hybrid	bind	hybrid	bind
4	domain	cell	cell	complex	cells	proteins	binds	interaction	binds	interaction
5	proteins	**interact**	proteins	**interact**	kinase	**interact**	identified	binds	analyse	binds
6	complex	cells	spots	proteins	domain	cell	analyse	analyse	complex	analyse
7	cell	domain	binds	cell	bind	complex	interaction	domain	interaction	expression
8	terminal	analyse	cells	hybrid	beta	cells	activation	human	activity	human
9	**interact**	complex	spot	protein	protein	kinase	function	activity	domain	activity
10	cells	interaction	domains	interacts	**interact**	receptor	activity	identified	identified	identified

**Table 4:** The top 10 features on the Data_BCIII_ selected by various feature selection methods. The terms that are selected commonly by all the methods are indicated in bold.

Number	GI	DF	CDM	Acc2	TFIDF	GINI_NTF_	GI_cs_	DF_cs_	CDM_cs_	Acc2_cs_
1	protein	protein	interacts	protein	cells	protein	hybrid	interact	binds	binds
2	bind	cell	**interaction**	bind	cell	cells	interact	binds	interact	interact
3	results	cells	interact	**interaction**	expression	cell	binds	study	bind	bind
4	cell	bind	binds	domain	**interaction**	**interaction**	expression	bind	expression	expression
5	cells	results	gene	complex	bind	expression	bind	expression	**interaction**	study
6	study	**interaction**	domain	proteins	protein	proteins	study	**interaction**	activity	**interaction**
7	**interaction**	activity	cell	kinase	proteins	complex	subunit	activity	complex	complex
8	using	proteins	terminal	gene	gene	domain	**interaction**	subunit	domain	activity
9	gene	study	interacting	cell	genes	gene	activity	results	results	terminal
10	use	function	ubiquitin	interacts	human	human	increase	complex	activity	results

**Table 5:** The comparison of common and special selected features on the Data_BCII_. *C* denotes the *F*1 measure of the Model_SVM_poly_ based on the common features selected by the frequency-based method and the context similarity-based method. The integer in parentheses is the number of the common features; CS denotes the *F*1 measure obtained by the context similarity-based method based on the top 1000 features. The number in parentheses is the increments compared with *C*; *F* denotes the *F*1 measure obtained by the frequency-based method based on the top 1000 features. The number in parentheses is the increments compared with *C*.

		GI_cs_	DF_cs_	CDM_cs_	Acc2_cs_
GI	*C*	71.62(714)	71.49(734)	71.40(702)	71.47(730)
	CS	76.64(+5.02)	76.57(+5.08)	76.81(+5.41)	76.60(+5.13)
	*F*	71.76(+0.14)	71.76(+0.27)	71.76(+0.36)	71.76(+0.29)

DF	*C*	72.31(625)	71.23(644)	71.06(613)	71.21(643)
	CS	76.64(+4.33)	76.57(+5.34)	76.81(+5.75)	76.60(+5.39)
	*F*	72.48(+0.17)	72.48(+1.25)	74.48(+3.42)	72.48(+1.27)

CDM	*C*	73.54(534)	73.17(552)	72.96(537)	73.16(552)
	CS	76.64(+3.10)	76.57(+3.40)	76.81(+3.85)	76.60(+3.44)
	*F*	74.92(+1.38)	74.92(+1.75)	74.92(+1.96)	74.92(+1.76)

Acc2	*C*	72.87(635)	73.40(650)	73.88(643)	73.40(650)
	CS	76.64(+3.77)	76.57(+3.17)	76.81(+2.93)	76.60(+3.20)
	*F*	74.04(+1.17)	74.04(+0.64)	74.04(+0.16)	74.04(+0.64)

TFIDF	*C*	72.90(668)	73.49(740)	73.40(692)	73.47(693)
	CS	76.64(+3.74)	76.57(+3.08)	76.81(+3.41)	76.60(+3.13)
	*F*	74.87(+1.97)	74.87(+1.38)	74.87(+1.47)	74.87(+1.40)

GINI_NTF_	*C*	73.67(720)	73.77(754)	73.49(710)	73.60(730)
	CS	76.64(+2.97)	76.57(+2.80)	76.81(+3.32)	76.60(+3.00)
	*F*	75.10(+1.43)	75.10(+1.33)	75.10(+1.61)	75.10(+1.50)

**Table 6:** The comparison of common and special selected features on the Data_BCIII_. *C* denotes the *F*1 measure of the Model_SVM_poly_ based on the common features selected by the frequency-based method and the context similarity-based method. The integer in parentheses is the number of the common features; CS denotes the *F*1 measure obtained by the context similarity-based method based on the top 1000 features. The number in parentheses is the increments compared with *C*; *F* denotes the *F*1 measure obtained by the frequency-based method based on the top 1000 features. The number in parentheses is the increments compared with *C*.

		GI_cs_	DF_cs_	CDM_cs_	Acc2_cs_
GI	*C*	55.22(740)	55.04(781)	55.54(764)	56.37(745)
	CS	58.78(+3.56)	58.76(+3.72)	59.09(+3.55)	58.57(+2.00)
	*F*	56.38(+1.16)	56.38(+1.34)	56.38(+0.84)	56.38(+0.01)

DF	*C*	57.02(579)	56.85(593)	56.70(658)	57.20(609)
	CS	58.78(+1.76)	58.76(+1.91)	59.09(+2.39)	58.57(+1.37)
	*F*	57.61(+0.59)	57.61(+0.76)	57.61(+0.91)	57.61(+0.41)

CDM	*C*	57.22(544)	57.11(545)	57.50(550)	57.18(560)
	CS	58.78(+1.56)	58.76(+1.65)	59.09(+1.59)	58.57(+1.39)
	*F*	57.09(−0.13)	57.09(−0.02)	57.09(−0.41)	57.09(−0.09)

Acc2	*C*	56.17(656)	56.13(678)	57.00(671)	56.51(673)
	CS	58.78(+2.61)	58.76(+2.63)	59.09(+2.09)	58.57(+2.06)
	*F*	57.09(+0.92)	57.09(+0.96)	57.09(+0.09)	57.09(+0.58)

TFIDF	*C*	57.20(668)	57.14(701)	57.05(692)	57.09(690)
	CS	58.78(+1.58)	58.76(+1.62)	59.09(+2.04)	58.57(+1.48)
	*F*	57.80(+0.60)	57.80(+0.66)	57.80(+0.75)	57.80(+0.71)

GINI_NTF_	*C*	57.35(720)	57.19(754)	57.17(715)	57.30(698)
	CS	58.78(+1.43)	58.76(+1.57)	59.09(+1.92)	58.57(+1.27)
	*F*	57.96(+0.61)	57.96(+0.77)	57.96(+0.79)	57.96(+0.66)

**Table 7:** Rate scores of dimension reduction on the Data_BCII_ and Data_BCIII_, respectively.

	GI	DF	CDM	Acc2	TFIDF	GINI_NTF_	GI_cs_	DF_cs_	CDM_cs_	Acc2_cs_
Data_BCII_	4640	4642	4664	4679	4693	4700	4729	4734	4738	4731
Data_BCIII_	2684	2667	2693	2695	2705	2712	2727	2723	2729	2728
